# Altered brain activity in patients with end‐stage renal disease: A meta‐analysis of resting‐state functional imaging

**DOI:** 10.1002/brb3.3057

**Published:** 2023-05-15

**Authors:** Wenjuan Song, Liuyan Zhao, Xuekun Li, Baolin Wu

**Affiliations:** ^1^ Department of Radiology First People's Hospital of Linping District Hangzhou China; ^2^ Department of Magnetic Resonance First Affiliated Hospital of Xinxiang Medical University Weihui China; ^3^ Huaxi MR Research Center (HMRRC), Department of Radiology West China Hospital of Sichuan University Chengdu China

**Keywords:** end‐stage renal disease, functional magnetic resonance imaging, meta‐analysis, resting state

## Abstract

**Introduction:**

: Previous studies have revealed abnormal resting‐state brain activity in patients with end‐stage renal disease (ESRD); however, the results are inconsistent. Thus, we conducted a coordinate‐based meta‐analysis of whole‐brain resting‐state functional neuroimaging studies in ESRD to identify the most consistent neural activity alterations in ESRD patients and explore their relation to serological indicators.

**Methods:**

: A comprehensive literature search strategy was applied to select pertinent studies up to December 2022 in PubMed, Web of Science, and Embase databases. Voxel‐wise meta‐analysis was conducted via the latest meta‐analytic algorithm, seed‐based *d* mapping with permutation of subject images software. Meta‐regression analyses were also conducted to explore the potential effect of clinical variables on resting‐state neural activity.

**Results:**

: Eleven studies comprising 304 patients with ESRD and 296 healthy controls (HCs) were included. Compared with HCs, ESRD patients showed decreased brain activity in the default mode network (DMN) regions, including the bilateral anterior cingulate cortex/medial prefrontal cortex, bilateral midcingulate cortex/posterior cingulate cortex, bilateral precuneus, and right angular gyrus. The neural activities in the bilateral midcingulate cortex, bilateral midcingulate cortex/posterior cingulate cortex, and right angular gyrus were significantly associated with serological indexes including hemoglobin, urea, and creatinine levels.

**Conclusion:**

: The present study provides a quantitative overview of brain activity alterations in patients with ESRD, and the results confirm the essential role of the DMN in ESRD patients, which may be the potential neural basis of their cognitive deficits. Additionally, some serological indicators may be used as predictive markers for progressive impairment of brain function.

## Graphical abstract

This quantitative coordinate‐based meta‐analysis of resting‐state functional imaging studies revealed impaired brain function in the default mode network regions in patients with end‐stage renal disease. Neural activities in some brain regions were associated with several serological indexes that related to renal function.

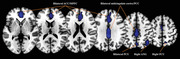

## 1 INTRODUCTION

Patients with chronic kidney disease (CKD) are at substantially higher risk for developing cognitive impairment compared with the general population (Drew et al., 2019). This risk is particularly true among dialysis end‐stage renal disease (ESRD) patients as a result of retention of uremic toxins, recurrent cerebral ischemia, and a high burden of inactivity (Shea et al., 2019). The prevalence of cognitive impairment in dialysis patients can be as high as 51% (San et al., 2017), and the cognitive domains involved mainly include orientation, attention, and executive function (O'Lone et al.,^2016^). Cognitive dysfunction in those patients is associated with poor outcomes and premature mortality (van Zwieten et al., 2019). Thus, a better understanding of the pathophysiology of cognitive impairment in patients with ESRD is essential for early intervention and treatment.

Resting‐state functional magnetic resonance imaging (rs‐fMRI) has provided a valuable tool to identify the neural mechanisms of cognitive impairment in patients with ESRD. Amplitude of low‐frequency fluctuation (ALFF) and regional homogeneity (ReHo) are the two commonly used methods to assess resting‐state brain activity. In theory, the ALFF is a reliable rs‐fMRI analytic algorithm to detect regional neural activity using the power spectrum of low‐frequency (0.01−0.08 Hz) fluctuations in the blood oxygen level–dependent signal (Zang et al., 2007), and the ReHo algorithm can explore local functional connectivity that measures the similarity of the resting‐state time series between one given voxel and its neighbor voxels (Zang et al., 2004). In the past decade, many rs‐fMRI studies using ReHo or ALFF analytic methods have revealed abnormal brain activity underlying cognitive deficits in patients with ESRD. Although previous rs‐fMRI studies have greatly improved our understanding of the pathophysiology of cognitive impairment in patients with ESRD, the findings from these studies have been less consistent than expected, and some results are even the opposite. For example, brain activity alterations in the sensorimotor areas (e.g., pre‐ and postcentral gyrus) in patients with ESRD relative to controls have been controversial. Decreased brain activity (Chen et al., 2015; Liang et al., 2013), increased brain activity (Chen et al., 2020), or null findings (Jin et al., 2020; Li et al., 2018) in these regions have been reported. Therefore, to overcome such inconsistency, it is necessary to conduct a quantitative meta‐analysis of previous rs‐fMRI studies on ESRD patients to identify reliable neurobiological markers.

Quantitative coordinate‐based meta‐analysis is a powerful and invaluable approach to quantifying voxel‐based neuroimaging findings (Tahmasian et al., 2019). In recent years, the techniques for coordinate‐based meta‐analysis have been evolving. As the latest meta‐analytic method, seed‐based *d* mapping with permutation of subject images (SDM‐PSI) can increase the accuracy of meta‐analytic results due to several strengths, such as controlling the family‐wise error rate (FWER) and using unbiased estimation of effect sizes, random‐effects models, Freedman–Lane‐based permutations, and threshold‐free cluster enhancement (TFCE) statistics (Albajes‐Eizagirre et al., 2019; Albajes‐Eizagirre et al., 2019; Albajes‐Eizagirre et al., 2019). This newly improved meta‐analytic method has been used to characterize brain abnormalities in patients with amnestic mild cognitive impairment (Zhang et al., 2021) and major depressive disorder (Zheng et al., 2021).

Thus, we aimed to conduct a meta‐analysis of all available rs‐fMRI studies on ESRD patients using the SDM‐PSI method to characterize the consistent changes in local brain activity. Additionally, meta‐regression analyses were performed to explore the potential effects of clinical features on resting‐state brain activity.

## 2 MATERIALS AND METHODS

### 2.1 Literature search

This meta‐analysis was performed based on the Preferred Reporting Items for Systematic Reviews and Meta‐Analysis (PRISMA) guidelines (Liberati et al., 2009) (Table S1). A comprehensive literature search strategy was applied to select pertinent studies up to December 2022 in PubMed, Web of Science, and Embase. The keywords “regional homogeneity,” “ReHo,” “amplitude of low‐frequency fluctuation,” “ALFF,” “fractional amplitude of low‐frequency fluctuation,” “fALFF,” “rest,” “resting,” “resting‐state,” “end‐stage renal disease,” and “ESRD” were used in both “AND” and “OR” combinations. Furthermore, the reference lists of the retrieved eligible articles and review articles were also searched manually to acquire additional relevant articles for inclusion.

### 2.2 Eligibility criteria

A study was included if it (1) was an original article published in a peer‐reviewed English‐language journal; (2) compared brain activity differences between patients with ESRD and healthy controls (HCs); (3) applied ALFF or fractional ALFF or ReHo algorithm to assess brain activity; and (4) clearly reported whole‐brain three‐dimensional Montreal Neurological Institute (MNI) or Talairach coordinates of the activated brain regions. A study was excluded if it was published as a case report, letter, abstract, review, or meta‐analysis or reported only specific region‐of‐interest findings.

### 2.3 Data extraction

For each included study, we extracted the following variables: (1) demographic data and clinical characteristics: sample size, age, sex, neuropsychological test results, and serological indexes. The extracted neuropsychological tests comprised number connection test‐A (NCT‐A), digit symbol test (DST), line‐tracing test (LTT), and serial‐dotting test (SDT), and the recorded serological indexes included serum creatinine (Scr), serum urea, and hemoglobin (Hb) levels. (2) Methodological information: field strength/MRI scanner, acquisition parameters, software packages for data analysis, smoothing kernel, statistical threshold, analytic method, and coordinate system.

### 2.4 Quality assessment

The qualities of the included studies were evaluated using a 10‐point checklist (Table S2), which was based on previous meta‐analysis studies of psychiatric disorders (Li et al., 2020; Ma et al., 2019). This checklist integrates the items regarding the sample characteristics and imaging‐specific methodology employed in the studies.

Two of the authors (W.J.S. and B.L.W.) independently performed the literature search, study selection, quality assessment, and data extraction. Any inconsistencies were discussed to reach a consensus.

### 2.5 Voxel‐wise meta‐analysis

This coordinate‐based meta‐analysis was performed using SDM‐PSI software (version 6.21; https://www.sdmproject.com/software). The analysis steps of SDM‐PSI have been described in detail in the SDM‐PSI tutorial (https://www.sdmproject.com/manual/) and previous studies (Albajes‐Eizagirre et al., 2019; Albajes‐Eizagirre et al., 2019), and are only briefly summarized here. First, the reported peak coordinates and corresponding effect sizes (derived, e.g., from *t* statistics) of clusters showing significant differences in brain activity between patients with ESRD and HCs were extracted from each dataset. Second, the maps of the lower and upper bounds of possible effect sizes were created for each dataset within a gray matter mask (anisotropy = 1, isotropic full width at half maximum = 20 mm, voxel size = 2 mm). Third, the maximum likelihood estimation technique was used to impute multiple effect‐size maps for each study, assuming that the effect sizes imputed follow a truncated normal distribution within the lower and upper bounds. Fourth, maps were combined in a standard random‐effects model considering sample size, intrastudy variability, and between‐study heterogeneity, and then these meta‐analysis images from the different imputed datasets were pooled using Rubin's rules (Albajes‐Eizagirre et al., 2019). Finally, we conducted family‐wise error correction for multiple comparisons and established statistical significance. In this meta‐analysis, we reported results using a corrected *p* < .05 (TFCE‐based FWER; 1000 permutations) with voxels extent ≥10.

### 2.6 Reliability analysis

To evaluate the reliability of our findings, systematic whole‐brain voxel‐based jackknife sensitivity analyses were performed by iteratively repeating the analysis leaving out one dataset each time (Radua & Mataix‐Cols, 2009; Radua et al., 2014).

### 2.7 Analysis of heterogeneity and publication bias

Heterogeneity between studies was assessed with the *I* ^2^ and *τ* ^2^ statistics using a random‐effects model. The *I* ^2^ statistic is a widely used measure of heterogeneity in meta‐analysis (Higgins & Thompson, 2002). An *I* ^2^ of 0% suggests that all variability in the findings is due to sampling error within studies. The following thresholds were used to interpret the level of heterogeneity: *I* ^2^ < 30%, mild; 30% < *I* ^2^ < 50%, moderate; *I* ^2^ > 50%, severe. However, *I* ^2^ reflects a proportion of heterogeneity but not an amount of heterogeneity, which means in some situations, the *I* ^2^ can be high when heterogeneity is low, and vice versa (Borenstein et al., 2017). Thus, it is not very helpful for measuring heterogeneity. In contrast, the *τ* ^2^ focuses on actual amounts of heterogeneity that can address the deficiency of *I* ^2^. For each significant cluster for an ESRD–HC comparison, we used funnel plots and Egger's test to assess the publication bias (an asymmetric plot and a *p* < .05 were considered significant).

### 2.8 Meta‐regression analysis

To investigate the potential effects of relevant clinical variables on main meta‐analysis results, we performed meta‐regression analyses by means of a simple linear regression. The clinical variable included mean age, percentage of male patients, disease duration, neuropsychological tests including NCT‐A, DST, LTT, and SDT, and serological indexes including Scr, urea, and Hb levels. The statistical threshold was set to *p* < .0005 (uncorrected) and voxels extent ≥10.

## 3 RESULTS

### 3.1 Included studies and sample characteristics

**FIGURE 1:**
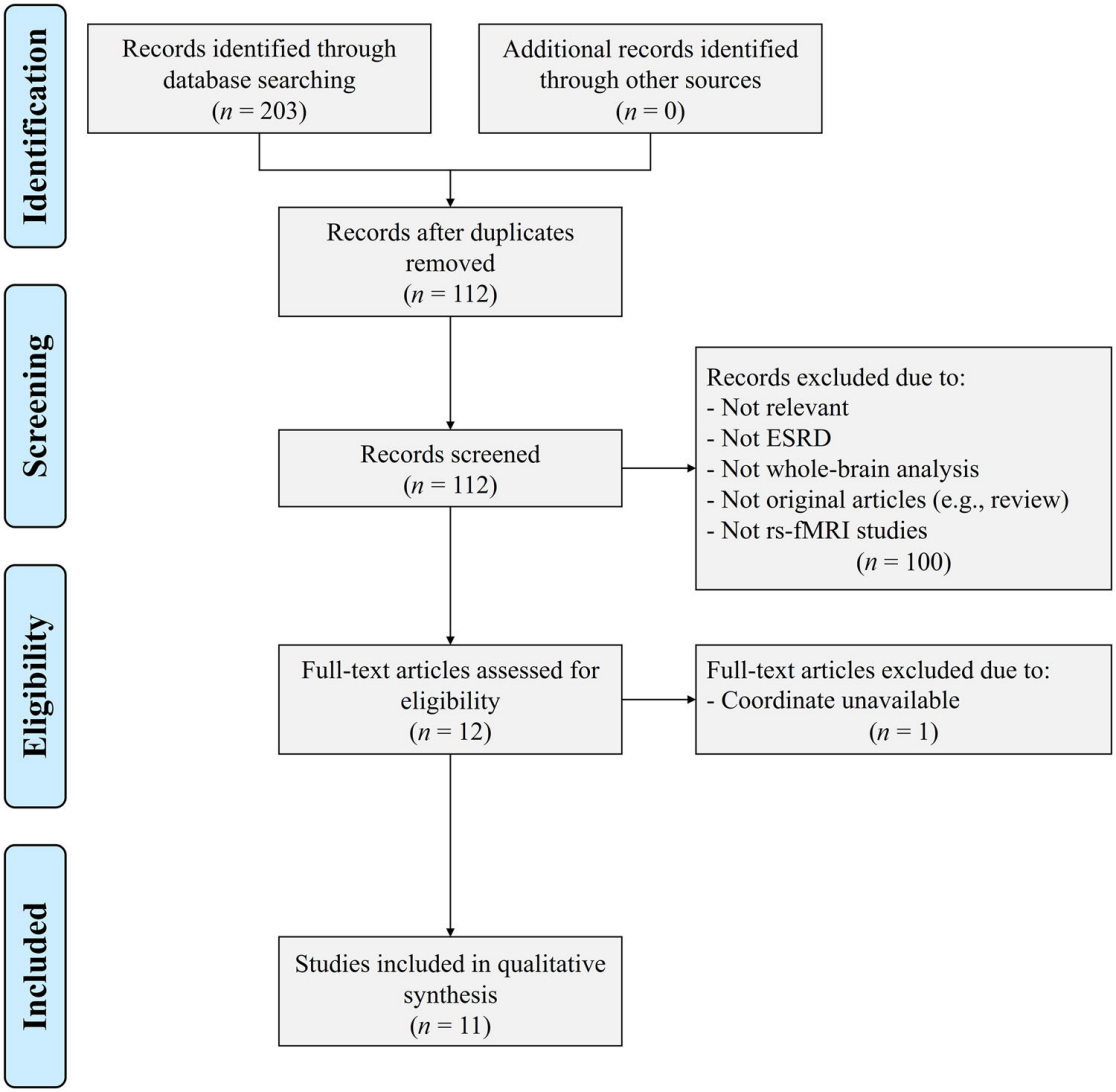
Flow chart of the identification of articles. ESRD, end‐stage renal disease; rs‐fMRI, resting‐state functional magnetic resonance imaging.

The detailed flow chart of study inclusion is shown in Figure 1. Eleven rs‐fMRI studies comprising 304 patients with ESRD (182 males and 122 females) and 296 HCs (172 males and 124 females) were included in the current meta‐analysis (Chen et al., 2015; Chen et al., 2020; Gu et al., 2020; Guo et al., 2021; Jin et al., 2020; Li et al., 2014; Li et al., 2018; Liang et al., 2013; Luo et al., 2016; Peng et al., 2021; Su et al., 2021). The percentage of males in the ESRD group and HC group was 59.87% and 58.11%, respectively, and there was no significant difference in gender ratio between the two groups (*X* ^2^ = 0.192, *p* = .661). No significant difference was found in the mean age between patients with ESRD (42.61 ± 8.20 years) and HCs (41.52 ± 8.31 years) (*t* = 0.310, *p* = .760, two‐sample *t*‐test). The detailed demographic and clinical characteristics are shown in Table 1, and the MR imaging technique details are summarized in Table S3.

**TABLE 1:** Demographic and clinical characteristics of subjects included in this meta‐analysis

	Number (male)		Mean age (SD), year		Neuropsychological tests, mean (SD)				Laboratory tests, mean (SD)				Quality scores (out of 10)
Studies	ESRD	HCs	ESRD	HCs	NCT‐A (s)	DST (score)	LTT (s)	SDT (s)	Scr (μmol/L)	Urea (mmol/L)	Hb (g/L)	Methods	
Liang et al. (2013)	19 (13)	20 (13)	37 (12.1)	36 (10.3)	61.1 (8.3)	41.5 (11.7)	68.7 (31.4)	60.3 (20.1)	811 (364.1)	23.1 (9.6)	NA	ReHo	9.5
Li et al. (2014)	20 (15)	20 (15)	37.1 (8.6)	38.3 (6.5)	NA	NA	NA	NA	NA	NA	NA	ReHo	9
Chen et al. (2015)	32 (22)	32 (22)	36.5 (9.6)	32.7 (8.8)	55.6 (15.7)	48.6 (15.1)	61.8 (22.6)	51.8 (10.5)	869.8 (278.4)	19.9 (8.8)	95.6 (20.6)	ReHo	9.5
Luo et al. (2016)	24 (16)	24 (16)	34 (8)	32 (9)	41 (14)	52 (12)	58 (22)	51 (16)	1182 (274)	26 (11)	86 (18)	ALFF	10
Li et al (2018)	23 (16)	25 (17)	34 (8.7)	33 (10.3)	NA	NA	NA	NA	937.4 (274.8)	23.4 (8.1)	103.6 (20.4)	ALFF	9.5
Gu et al. (2020)	35 (15)	25 (13)	47.6 (6.1)	47.7 (5.2)	NA	NA	NA	NA	NA	NA	NA	ALFF	9
Jin et al. (2020)	46 (28)	47 (22)	53.1 (1.6)	55.6 (0.9)	NA	NA	NA	NA	935 (222.4)	NA	114.3 (12.2)	ALFF	10
Chen et al. (2020)	19 (6)	17 (9)	45.2 (6.7)	41.8 (9.9)	44.2 (32.9)	36 (11.9)	55.4 (24.7)	44.7 (25.4)	967.3 (209.1)	22.1 (7.2)	128.5 (24.3)	ALFF	10
Peng et al. (2021)	24 (14)	27 (10)	56.2 (11.9)	50.7 (11.2)	78.7 (59.2)	35.4 (14.5)	69.8 (23.4)	56.8 (12.6)	NA	NA	NA	ALFF	9
Guo et al. (2021)	42 (22)	42 (22)	50.9 (10.2)	50.4 (10)	NA	NA	NA	NA	NA	NA	108.1 (14.6)	ALFF	9
Su et al. (2021)	20 (15)	17 (13)	37.1 (8.6)	38.5 (6.9)	46.8 (13.4)	47.2 (9.7)	45.1 (12.1)	44.3 (9.4)	1038.6 (250.5)	21.6 (4.2)	98.4 (19.8)	ALFF	9

Abbreviations: ALFF, amplitude of low‐frequency fluctuations; DST, digital symbol test; ESRD, end‐stage renal disease; Hb, hemoglobin; HCs, healthy controls; LTT, line‐tracing test; NA, not available; NCT‐A, number connection test‐A; ReHo, regional homogeneity; Scr, serum creatinine; SD, standard deviation; SDT, serial‐dotting test.

### 3.2 Main meta‐analysis

**FIGURE 2:**
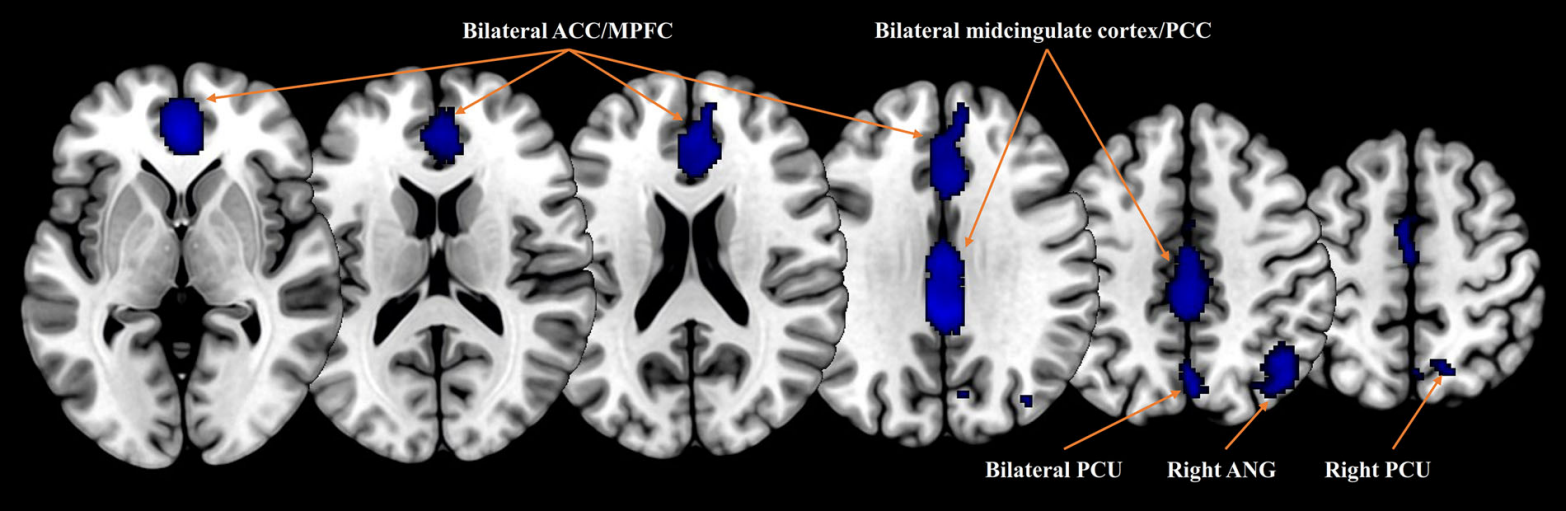
Brain regions of decreased resting‐state brain activity in patients with ESRD compared with healthy controls. ACC, anterior cingulate cortex; MPFC, medial prefrontal cortex; PCC, posterior cingulate cortex; PCU, precuneus; ANG, angular gyrus.

Compared with HCs, patients with ESRD displayed decreased resting‐state brain activity in the default mode network (DMN) regions, including the bilateral anterior cingulate cortex/medial prefrontal cortex, bilateral posterior cingulate cortex/midcingulate cortex, bilateral precuneus, and right angular gyrus (*p* < .05, TFCE FWER‐corrected) (Table 2; Figure 2). Whole‐brain jackknife sensitivity analyses revealed that the main results were highly replicable, as decreased brain activity in the bilateral anterior cingulate cortex/medial prefrontal cortex, right angular gyrus, and left precuneus remained significant in all combinations of the datasets and decreased brain activity in the bilateral midcingulate cortex/posterior cingulate cortex and right precuneus remained significant in all but one combination of the datasets (Table S4).

**TABLE 2:** Brain regions showing reduced resting‐state brain activity in patients with ESRD compared with HCs

Brain regions	MNI coordinates					Cluster	
	*x*	*y*	*z*	SDM z‐score	*p* value corrected	No. of voxels^a^	Cluster breakdown (no. of voxels)
Bilateral anterior cingulate cortex/medial prefrontal cortex, BA 10	0	44	0	−5.120	0.009999990	2063	Right anterior cingulate cortex, BA 10, 11, 24, 25, 32 (516) Left anterior cingulate cortex, BA 11, 24, 25, 32 (682) Right medial part of superior frontal gyrus, BA 9, 10, 11, 32 (313) Left medial part of superior frontal gyrus, BA 10, 11, 24, 32 (281) Right midcingulate cortex, BA 9, 24, 32 (130) Left midcingulate cortex, BA 24, 32 (74)
Bilateral midcingulate cortex/posterior cingulate cortex, BA 23	2	−28	38	−6.122	∼0	1700	Right midcingulate cortex, BA 23 (448) Left midcingulate cortex, BA 23, 24 (459) Left posterior cingulate cortex, BA 23, 26 (70) Right posterior cingulate cortex, BA 23, 26 (33)
Right angular gyrus, BA 39	46	−64	42	−4.657	0.009999990	440	Right angular gyrus, BA 7, 19, 39, 40 (296) Right middle occipital gyrus, BA 7, 19, 39 (93) Right inferior parietal gyri, BA 39, 40 (25)
Left precuneus, BA 7	2	−70	44	−4.470	0.019999981	164	Right precuneus, BA 7 (88) Left precuneus, BA 7 (41) Right cuneus cortex, BA 7, 19 (24)
Right precuneus, BA 5	12	−60	58	−3.977	0.029999971	29	Right precuneus, BA 5, 7 (11) Right superior parietal gyrus, BA 5, 7 (11)

Abbreviations: BA, Brodmann area; ESRD, end‐stage renal disease; HCs, healthy controls; L, left; MNI, Montreal Neurological Institute; R, right; SDM, seed‐based *d* mapping.

^a^ All voxels with *p* < .05 (family‐wise error rate corrected).

### 3.3 Quality assessment, heterogeneity, and publication bias analyses

The mean score for quality assessment was 9.4 out of 10 points (range: 9–10 points) (Table 1). The main results exhibited mild interstudy variability after heterogeneity analyses (Table S5). Egger's tests were nonsignificant for all clusters in the main meta‐analysis (all *p* > .05; Table S5), suggesting that there was no publication bias.

### 3.4 Meta‐regression analysis results

**FIGURE 3:**
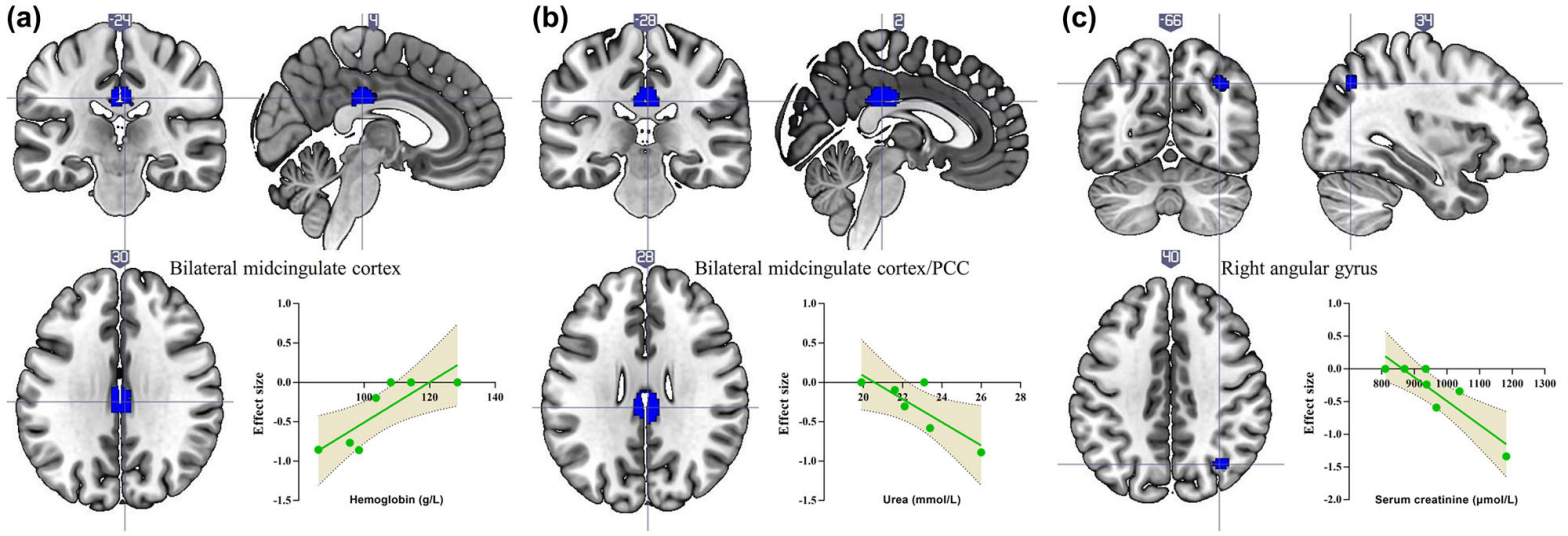
Meta‐regression analyses show a positive association between hemoglobin level and brain activity in the bilateral midcingulate cortex (a), a negative association between urea level and brain activity in the bilateral midcingulate cortex/posterior cingulate cortex (PCC) (b), and a negative association between serum creatinine level and brain activity in the right angular gyrus (c). Each study is presented as a dot. The regression line (meta‐regression signed differential mapping slope) is presented as a straight line. The shaded areas represent 95% confidence interval.

Figure 3 illustrates the meta‐regression analysis results. The mean Hb level (available from seven datasets) was positively associated with resting‐state brain activity in the bilateral midcingulate cortex (voxels = 185; MNI coordinates: *x* = 4, *y* = −24, *z* = 30; SDM *z*‐score = 3.705; *p* = .0001, uncorrected; Brodmann area [BA] 23). The mean urea level (available from six datasets) was negatively associated with resting‐state brain activity in the bilateral midcingulate cortex/posterior cingulate cortex (voxels = 242; MNI coordinates: *x* = 2, *y* = −28, *z* = 28; SDM *z*‐score = −4.149; *p* = .00002, uncorrected; BA 23). The mean Scr level (available from seven datasets) was negatively associated with resting‐state brain activity in the right angular gyrus (voxels = 38; MNI coordinates: *x* = 34, *y* = −66, *z* = 40; SDM *z*‐score = −3.929; *p* = .00004, uncorrected; BA 7). No effects of mean age (available from all datasets), percentage of male patients (available from all datasets), and neuropsychological test results (available from 6 datasets) were detected, at least linearly.

## 4 DISCUSSION

The present voxel‐wise meta‐analysis provides a unique opportunity to characterize resting‐state brain activity abnormalities in patients with ESRD. To our knowledge, this is the first coordinate‐based meta‐analysis to identify the patterns of changes in resting‐state brain activity in patients with ESRD compared with HCs. Our study identified that the consistent brain regions with decreased brain activity in patients with ESRD were mainly located in the DMN. In addition, brain activities in the bilateral cingulate cortex, bilateral cingulate cortex/posterior cortex, and right angular gyrus were associated with the serological indexes including Scr, Hb, and urea levels in patients with ESRD.

### 4.1 Reduced resting‐state brain activity in the DMN

As one of the most important resting‐state brain networks, the DMN is engaged in the maintenance of the baseline brain activities associated with cognition of self‐awareness, episodic memory, and interactive modulation between the internal mind activities and external tasks (Buckner et al., 2008; Greicius et al., 2003; Gusnard et al., 2001). Similarly, decreased cortical thickness (Dong et al., 2018), gray matter volume (Zhang et al., 2013), and functional connectivity (Ni et al., 2014) in the DMN regions were also found in patients with ESRD. Moreover, recent graph theory–based studies revealed decreased nodal centralities in some DMN regions (Chouet al., 2019; Jin et al., 2021; Yue et al., 2021), such as the anterior cingulate cortex, right angular gyrus, and dorsal medial prefrontal cortex. Thus, our meta‐analysis further confirmed DMN dysfunction in patients with ESRD, which may be the potential neural basis of cognitive declines in patients with ESRD. Although we did not find significant associations between brain activities in the DMN regions and neuropsychological test results, several previous studies have demonstrated that the cognitive impairment in patients with ESRD was associated with gray matter volume deficits in the posterior cingulate cortex, precuneus, and anterior cingulate cortex (Zhang et al., 2013) and reduced functional connectivity in the medial frontal cortex (Ni et al., 2014). These nonsignificant meta‐regression analysis results may be attributed to the unavailability of neuropsychological test data in some datasets.

### 4.2 Effects of serological indexes on resting‐state brain activity

Meta‐regression analysis revealed that the Hb level was positively correlated with brain activity in the bilateral midcingulate cortex, a core region contributing to cognitive control (Huster et al., 2014). Due to a decrease in erythropoietin secretion levels resulting from the reduction of kidney function, anemia is common in CKD patients, and the prevalence of anemia is strongly associated with a declining estimated glomerular filtration rate (McClellan et al., 2004). Anemia can lead to an increase in cerebral blood flow and thus contributes to an increase in uremic toxin content in brain tissues, resulting in extensive damage to the brain microstructure, which may be associated with cognitive impairment in patients with ESRD (Jiang et al., 2016; Kurella Tamura et al., 2016). This assumption was also supported by a prior clinical trial demonstrating that an increase in Hb levels after erythropoietin treatment was associated with an improvement in cognitive function (Marsh et al., 1991). Thus, our meta‐analysis further supports the notion that cognitive improvement can benefit from anemia treatment in patients with ESRD. A previous functional neuroimaging study found that lower hematocrit levels affected functional connectivity in patients with ESRD (Zheng et al., 2014). Another neuroimaging study investigated the structural and functional alterations of the DMN after renal transplantation in patients with ESRD, and found that hematocrit level was positively associated with changes in mean diffusivity of the fiber bundles connecting the posterior cingulate cortex/precuneus to the right inferior parietal lobule. Together, these findings highlight the impact of anemia on brain structure and function in patients with ESRD.

We found that the urea and Scr levels were negatively correlated with brain activity in the bilateral midcingulate cortex/posterior cingulate cortex and right angular gyrus, respectively. Due to direct or indirect effects, uremic toxins may contribute to a central nervous system injury (Arnold et al., 2016). On one hand, many uremic toxins are likely to play an important role directly resulting in neurotoxicity; on the other hand, the uremic milieu in patients with ESRD can cause indirect damage to the brain tissue through their contribution to systemic inflammation, endothelial dysfunction, and atherosclerosis (Arnold et al., 2016). In line with our findings, Ni et al. (2014) found that the Scr level of patients with ESRD negatively correlated with functional connectivity of the bilateral posterior cingulate cortex and precuneus in the DMN. Thus, we speculate that the Scr and urea levels may play an important role in posterior DMN dysfunction in patients with ESRD.

### 4.3 Limitations

We acknowledge several limitations in this meta‐analysis. First, the number of included studies was relatively small, which may affect the power of the statistical analysis. Second, the heterogeneity of the selected studies could not be avoided due to the differences in demographics, imaging parameters, and analytical methods. Third, we could not assess the potential effects of dialysis and different dialysis modalities (hemodialysis and peritoneal dialysis) on brain activity due to a small number of included studies. Further comparative meta‐analyses should be conducted in the future as more relevant rs‐fMRI studies are available.

## 5 CONCLUSION

In summary, this is the first meta‐analysis to characterize the patterns of change in resting‐state brain activity in patients with ESRD using a novel meta‐analytic method. We identified that the most consistent and reliable regions with abnormal brain activity were located at the DMN. In addition, brain activities in the midcingulate cortex/posterior cortex and right angular gyrus were associated with Scr, urea, and Hb levels, suggesting that these indicators may be used as predictive markers for progressive impairment of brain function.

## AUTHOR CONTRIBUTIONS

Baolin Wu and Wenjuan Song contributed to the conception and design of the study. Baolin Wu, Wenjuan Song, Liuyan Zhao, and Xuekun Li contributed to literature search, data extraction, data analysis, and data interpretation. Baolin Wu and Wenjuan Song contributed to the drafting of the manuscript. Xuekun Li obtained funding to support this work. Baolin Wu critically revised the manuscript. All authors approved the final version of the manuscript.

## CONFLICT OF INTEREST STATEMENT

The authors declare no conflicts of interest.

### PEER REVIEW

The peer review history for this article is available at https://publons.com/publon/10.1002/brb3.3057.

## Supporting information

TABLE S1. PRISMA checklist
TABLE S2. Quality assessment checklist (when criteria were partially met, 0.5 points were assigned).
TABLE S3. Technique details of resting‐state fMRI studies included in the meta‐analysis.
TABLE S4. Jackknife sensitivity analyses of resting‐state fMRI studies included in the meta‐analysis
Table S5. Analysis of heterogeneity and publication bias.

## Data Availability

The data that support the findings of this study are available from the corresponding author upon reasonable request.
