# Supplementary material for: Altered brain activity in patients with end‐stage renal disease: A meta‐analysis of resting‐state functional imaging
Source: Brain Behav. 2023 May 15;13(7):e3057. doi: 10.1002/brb3.3057 (PMC10338797; doi:10.1002/brb3.3057)
Supplement: Supplementary file 1 — TABLE S1. PRISMA checklist TABLE S2. Quality assessment checklist (when criteria were partially met, 0.5 points were assigned). TABLE S3. Technique details of resting‐state fMRI studies included in the meta‐analysis. TABLE S4. Jackknife sensitivity analyses of resting‐state fMRI studies included in the meta‐analysis Table S5. Analysis of heterogeneity and publication bias. [file BRB3-13-e3057-s001.docx]

**Supplementary Materials**

**Altered brain activity in patients with end-stage renal disease: a meta-analysis of resting-state functional imaging**

**TABLE S1.** PRISMA checklist

**TABLE S2.** Quality assessment checklist (when criteria were partially met, 0.5 points were assigned).

**TABLE S3.** Technique details of resting-state fMRI studies included in the meta-analysis.

**TABLE S4.** Jackknife sensitivity analyses of resting-state fMRI studies included in the meta-analysis

**Table S5.** Analysis of heterogeneity and publication bias.

**TABLE S1. PRISMA checklist**

| **Section / topic** | **#** | **Checklist item** | **Reported on page #** |
| --- | --- | --- | --- |
| **TITLE** | | |  |
| Title | 1 | Identify the report as a systematic review, meta-analysis, or both. | 1 |
| **ABSTRACT** | | |  |
| Structured summary | 2 | Provide a structured summary including, as applicable: background; objectives; data sources; study eligibility criteria, participants, and interventions; study appraisal and synthesis methods; results; conclusions and implications of key findings. | 2 |
| **INTRODUCTION** | | |  |
| Rationale | 3 | Describe the rationale for the review in the context of what is already known. | 4-5 |
| Objectives | 4 | Provide an explicit statement of questions being addressed with reference to participants, interventions, comparisons, outcomes, and study design (PICOS). | 6 |
| **METHODS** | | |  |
| Protocol and registration | 5 | Indicate if a review protocol exists, if and where it can be accessed (e.g., Web address), and, if available, provide registration information including registration number. | NA |
| Eligibility criteria | 6 | Specify study characteristics (e.g., PICOS, length of follow-up) and report characteristics (e.g., years considered, language, publication status) used as criteria for eligibility, giving rationale. | 6-7 |
| Information sources | 7 | Describe all information sources (e.g., databases with dates of coverage, contact with study authors to identify additional studies) in the search and date last searched. | 7 |
| Search | 8 | Present full electronic search strategy for at least one database, including any limits used, such that it could be repeated. | 6 |
| Study selection | 9 | State the process for selecting studies (i.e., screening, eligibility, included in systematic review, and, if applicable, included in the meta-analysis). | Fig. 1 |
| Data collection process | 10 | Describe method of data extraction from reports (e.g., piloted forms, independently, in duplicate) and any processes for obtaining and confirming data from investigators. | 7 |
| Data items | 11 | List and define all variables for which data were sought (e.g., PICOS, funding sources) and any assumptions and simplifications made. | Table 1 |
| Risk of bias in individual studies | 12 | Describe methods used for assessing risk of bias of individual studies (including specification of whether this was done at the study or outcome level), and how this information is to be used in any data synthesis. | 8-9 |
| Summary measures | 13 | State the principal summary measures (e.g., risk ratio, difference in means). | 8-9 |
| Synthesis of results | 14 | Describe the methods of handling data and combining results of studies, if done, including measures of consistency (e.g., I^2^) for each meta-analysis. | 9 |
| Risk of bias across studies | 15 | Specify any assessment of risk of bias that may affect the cumulative evidence (e.g., publication bias, selective reporting within studies). | 9 |
| Additional analyses | 16 | Describe methods of additional analyses (e.g., sensitivity or subgroup analyses, meta-regression), if done, indicating which were pre-specified. | 9 |
| **RESULTS** | | |  |
| Study selection | 17 | Give numbers of studies screened, assessed for eligibility, and included in the review, with reasons for exclusions at each stage, ideally with a flow diagram. | 10 |
| Study characteristics | 18 | For each study, present characteristics for which data were extracted (e.g., study size, PICOS, follow-up period) and provide the citations. | 10 |
| Risk of bias within studies | 19 | Present data on risk of bias of each study and, if available, any outcome level assessment (see item 12). | 11 |
| Results of individual studies | 20 | For all outcomes considered (benefits or harms), present, for each study: (a) simple summary data for each intervention group (b) effect estimates and confidence intervals, ideally with a forest plot. | NA |
| Synthesis of results | 21 | Present results of each meta-analysis done, including confidence intervals and measures of consistency. | 11 |
| Risk of bias across studies | 22 | Present results of any assessment of risk of bias across studies (see Item 15). | 11 |
| Additional analysis | 23 | Give results of additional analyses, if done (e.g., sensitivity or subgroup analyses, meta-regression [see Item 16]). | 11 |
| **DISCUSSION** | | |  |
| Summary of evidence | 24 | Summarize the main findings including the strength of evidence for each main outcome; consider their relevance to key groups (e.g., healthcare providers, users, and policy makers). | 12-15 |
| Limitations | 25 | Discuss limitations at study and outcome level (e.g., risk of bias), and at review-level (e.g., incomplete retrieval of identified research, reporting bias). | 15 |
| Conclusions | 26 | Provide a general interpretation of the results in the context of other evidence, and implications for future research. | 15 |
| **FUNDING** | | |  |
| Funding | 27 | Describe sources of funding for the systematic review and other support (e.g., supply of data); role of funders for the systematic review. | 15 |

**TABLE S2.** Quality assessment checklist (when criteria were partially met, 0.5 points were assigned)

| **Category 1: Participants** | Score (0/0.5/1) |
| --- | --- |
| 1. Patients were evaluated prospectively, specific diagnostic criteria were applied, and demographic data were reported.  2. Comparison participants were evaluated prospectively, with psychiatric and medical illnesses were excluded.  3. Important variables (e.g., age, sex, illness duration, onset, medication status, BMI, HbA1c, intelligence quotient, i.e. IQ, handedness) were checked either by stratification or statistically.  4. Sample size per group > 10. | |
| **Category 2: Methods for image acquisition and analysis** | |
| 5. Whole brain analysis was automated with no a priori regional selection.  6. Coordinates reported in a standard space.  7. The imaging technique used was clearly described so that it could be reproduced.  8. Measurements were clearly described so that they could be reproduced. | |
| **Category 3: Results and conclusions** | |
| 9. Statistical parameters for significant and important non-significant differences were provided.  10. Conclusions were consistent with the results obtained and the limitations were discussed. | |
| TOTAL /10 | |

| Datasets | Field strength/MRI scanner | Head coil | TR/TE (ms) | Voxel size (mm^3^) | Software | FWHM  (mm) | Statistical threshold | Covariates | Coordinate  system |
| --- | --- | --- | --- | --- | --- | --- | --- | --- | --- |
| Liang et al. (2013) | 3.0 T Siemens | NA | 2000/30 | Slice thickness = 4 mm^*^ | SPM8, REST | 8 | *p* < 0.05 (AlphaSim) | Age and gender | MNI |
| Li et al. (2014) | 1.5 T Philips | NA | 3000/50 | Slice thickness = 4.5 mm^*^ | DPARSF | 8 | *p* < 0.05 (AlphaSim) | Age and education | MNI |
| Chen et al. (2015) | 3.0 T Siemens | NA | 2000/30 | Slice thickness = 4 mm^*^ | SPM8 | 8 | *p* < 0.05 (AlphaSim) | Age and gender | MNI |
| Luo et al. (2016) | 3.0 T Siemens | 12-channel | 2000/30 | Slice thickness = 4 mm^*^ | SPM8, DPARSF | NA | *p* < 0.01 (AlphaSim) | Age and gender | MNI |
| Li et al (2018) | 3.0 T GE | 8-channel | 2000/50 | 3 × 3 × 3 | SPM8, DPARSF | 6 | *p* < 0.001 (FDR) | Age and gender | MNI |
| Gu et al. (2020) | 3.0 T Philips | 16-channel | 2000/30 | Slice thickness = 4 mm^*^ | DPABI | 4 | *p* < 0.05 (GRF) | Age, gender and education | MNI |
| Jin et al. (2020) | 3.0 T GE | 8-channel | 2000/35 | Slice thickness = 5.0 mm^*^ | DPARSF | 6 | *p* < 0.05 (FDR) | Age and gender | MNI |
| Chen et al. (2020) | 3.0 T Siemens | NA | 2000/30 | Slice thickness = 3.5 mm^*^ | DPARSF | 4 | *p* < 0.05 (GRF) | Age, gender and education | MNI |
| Peng et al. (2021) | 1.5 T Siemens | 8-channel | 4170/50 | Slice thickness = 5.0 mm^*^ | RESTplus | 6 | *p* < 0.05 (FDR) | Age, gender and education | MNI |
| Guo et al. (2021) | 3.0 T GE | NA | 2000/30 | Slice thickness = 3.5 mm^*^ | DPABI | NA | *p* < 0.05 (AlphaSim) | Age and gender | MNI |
| Su et al. (2021) | 3.0 T Philips | NA | 2000/30 | Slice thickness = 3.5 mm^*^ | DPARSF | 4 | *p* < 0.05 (FDR) | Age, gender and education | MNI |

**TABLE S3.** Technique details of resting-state fMRI studies on patients with ESRD included in this meta-analysis

Abbreviations: FDR = false discovery rate; GRF = Gaussian random field; MNI = Montreal Neurological Institute; TR/TE = repetition time/echo time; FWHM = full width half maximum; NA = not available.

^*^ Details are not available.

| **Discarded dataset** | **ESRD < HCs** | | | | |
| --- | --- | --- | --- | --- | --- |
|  | Bilateral ACC/MPFC | Bilateral midcingulate cortex/PCC | Right angular gyrus | Left precuneus | Right precuneus |
| Liang et al. (2013) | Y | Y | Y | Y | N |
| Li et al. (2014) | Y | Y | Y | Y | Y |
| Chen et al. (2015) | Y | Y | Y | Y | Y |
| Luo et al. (2016) | Y | N | Y | Y | Y |
| Li et al (2018) | Y | Y | Y | Y | Y |
| Gu et al. (2020) | Y | Y | Y | Y | Y |
| Jin et al. (2020) | Y | Y | Y | Y | Y |
| Chen et al. (2020) | Y | Y | Y | Y | Y |
| Peng et al. (2021) | Y | Y | Y | Y | Y |
| Guo et al. (2021) | Y | Y | Y | Y | Y |
| Su et al. (2021) | Y | Y | Y | Y | Y |
| Total | 11/11 | 10/11 | 11/11 | 11/11 | 10/11 |

**TABLE S4.** Jackknife sensitivity analyses of resting-state fMRI studies included in the meta-analysis

Abbreviations: ESRD = end-stage renal disease; HCs = healthy controls; ACC = anterior cingulate cortex; MPFC = medial prefrontal cortex; PCC = posterior cingulate cortex; Y = yes; N = no

**TABLE S5.** Analysis of heterogeneity and publication bias

| Brain areas | MNI coordinates | Funnel plots | Egger test | Heterogeneity analysis | |
| --- | --- | --- | --- | --- | --- |
|  | (x, y, z) |  |  | *I*^2^ | *Tau*^2^ |
| Bilateral anterior cingulate cortex/medial prefrontal cortex | 0, 44, 0 | 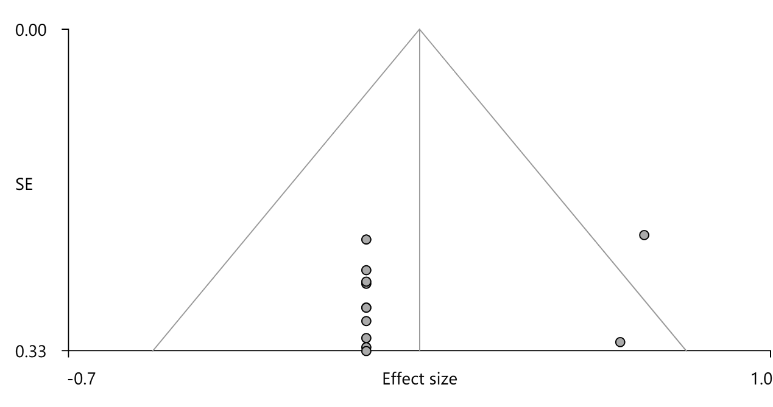 | *p* = 0.338 | 0.6236 | 0.0006 |
| Bilateral midcingulate cortex/posterior cingulate cortex | 2, -28, 38 | 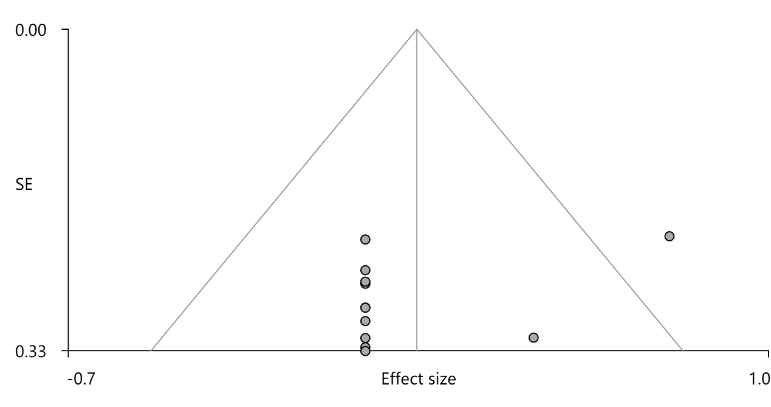 | *p* = 0.191 | 8.7786 | 0.0103 |
| Right angular gyrus | 46, -64, 42 | 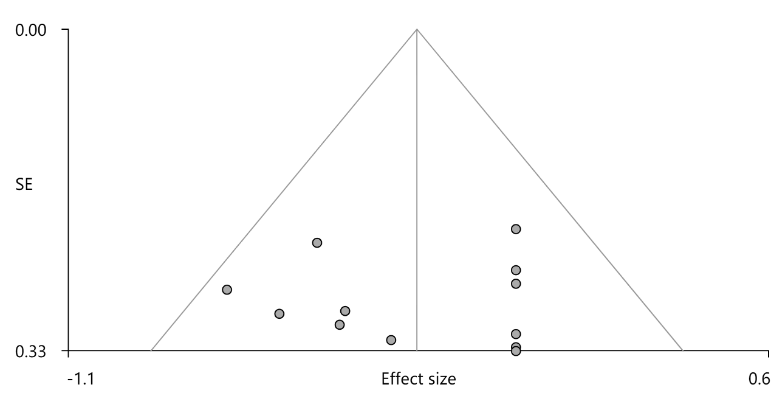 | *p* = 0.895 | 1.8896 | 0.0018 |
| Left precuneus | 2, -70, 44 | 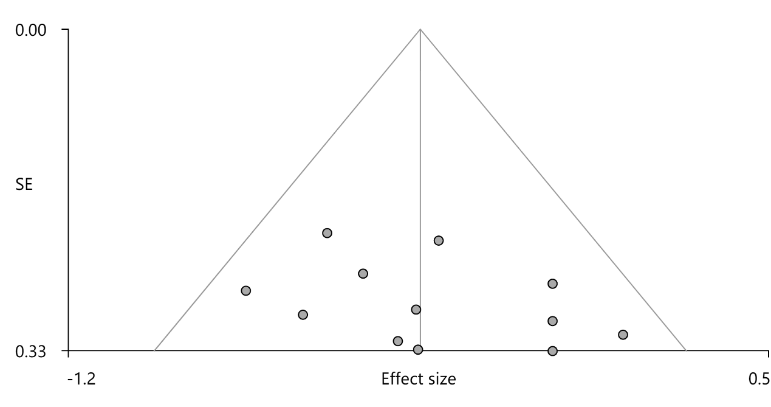 | *p* = 0.224 | 1.2160 | 0.0011 |
| Right precuneus | 12, -60, 58 | 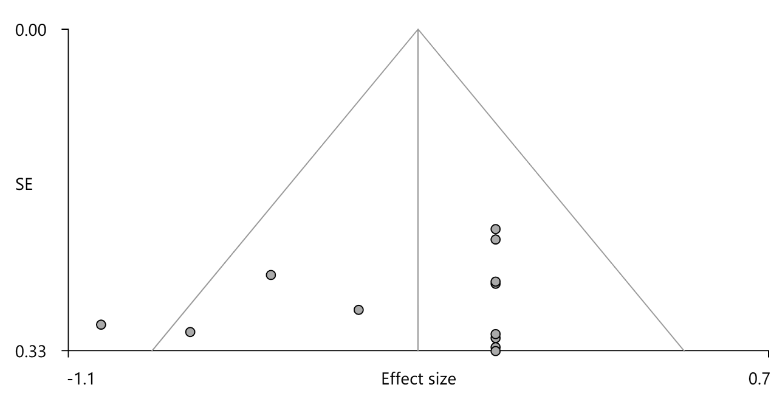 | *p* = 0.367 | 0.4669 | 0.0005 |
